# Caries status in 12‐year‐old children, geographical location and socioeconomic conditions across European countries: A systematic review and meta‐analysis

**DOI:** 10.1111/ipd.13224

**Published:** 2024-06-16

**Authors:** Ana Vukovic, Kian Alessandro Schmutz, Roberta Borg‐Bartolo, Fabio Cocco, Ruxandra Sava Rosianu, Rainer Jorda, Anastasia Maclennon, Javier F. Cortes‐Martinicorenas, Christos Rahiotis, Melinda Madléna, Antonella Arghittu, Marco Dettori, Paolo Castiglia, Marcella Esteves‐Oliveira, Maria Grazia Cagetti, Thomas G. Wolf, Guglielmo Campus

**Affiliations:** ^1^ Department of Pediatric and Preventive Dentistry, School of Dental Medicine University of Belgrade Belgrade Serbia; ^2^ Department of Restorative, Preventive and Pediatric Dentistry University of Bern Bern Switzerland; ^3^ Department of Surgery, Microsurgery and Medicine Sciences, School of Dentistry University of Sassari Sassari Italy; ^4^ Department of Preventive, Community Dentistry and Oral Health, Faculty of Dentistry University of Medicine and Pharmacy “Victor Babes” Timisoara Romania; ^5^ Institute of German Dentists (IDZ) Cologne Germany; ^6^ Ex‐Facultad de Odontología Universidad de Barcelona Pamplona Spain; ^7^ Department of Operative Dentistry National and Kapodistrian University of Athens Athens Greece; ^8^ Department of Community Dentistry, Faculty of Dentistry Semmelweis University Budapest Hungary; ^9^ Department of Conservative Dentistry, Periodontology and Endodontology, University Centre of Dentistry, Oral Medicine and Maxillofacial Surgery (UZMK) University of Tübingen Tübingen Germany; ^10^ Department of Biomedical, Surgical and Dental Sciences University of Milan Milan Italy; ^11^ Department of Periodontology and Operative Dentistry University of Mainz Mainz Germany; ^12^ Department of Cariology Saveetha Dental College and Hospitals, SIMATS Chennai India

**Keywords:** caries epidemiology, children, Europe, healthcare systems, socioeconomic indicators

## Abstract

**Background:**

Understanding of socioeconomic context might enable more efficient evidence‐based preventive strategies in oral health.

**Aim:**

The study assessed the caries‐related socioeconomic macro‐factors in 12‐year‐olds across European countries.

**Design:**

This systematic review involved epidemiological surveys on the caries status of 12‐year‐olds from 2011 to 2022. DMFT was analyzed in relation to gross national income (GNI), United Nations Statistical Division geographical categorization of European countries (M49), unemployment rate, Human Development Index (HDI), and per capita expenditure on dental health care. A meta‐analysis was performed for countries reporting data on DMFT, stratified by GNI, and geographical location of European countries, using a random‐effects model.

**Results:**

The study involved 493 360 children from 36 countries in the geographic region of Europe. The analysis confirmed a strong negative correlation between income and caries experience (*p* < .01). Children living in higher‐income countries showed 90% lower odds of poor oral health than in middle‐income countries. Children living in West Europe showed 90% lower odds of poor oral health than children living in East Europe.

**Conclusion:**

The strong effect of macro‐level socioeconomic contexts on children's oral health suggests favoring upstream preventive oral health strategies in countries with economic growth difficulties, Eastern and Southern parts of Europe.

**Why this paper is important for paediatric dentists**

- This systematic review and meta‐analysis of studies from European countries show caries experience ranging from 0.30 in Denmark to 6.88 in North Macedonia.
- Caries experience was statistically associated with socioeconomic indicators such as type of geographical location, GNI, and employment rate, with a suggestion of responsible structural or methodological components.
- Of 44 European countries, caries data in 12‐year‐old children were retrieved for 36 countries and this might be a bias influencing the result of this review.

## 1 INTRODUCTION

Dental caries as noncommunicable disease (NCD) shares with other NCDs' modifiable risk factors such as high sugar intake and poor health behavior; its prevalence is strongly influenced by socioeconomic contexts.^1^ These determinants strongly influence stark oral health inequalities between and within countries, creating resource unavailability, patient vulnerability and poor accessibility to health care in underprivileged social groups.^2^ According to the conceptual theoretical framework proposed by the World Health Organization (WHO) Commission on social determinants of health, the macro‐level context influencing health outcomes involves several items as follows: (1) governance, (2) macroeconomic policies, (3) social policies, (4) public policies, (5) cultural and societal values, and (6) epidemiological circumstances.^3^

Social determinants at the micro‐level (individual interaction) and meso‐level (community interaction) have been identified as strong risk factors contributing to poor oral health.^4^ Until now, strategies affecting micro‐level modifiable factors were not sufficient to improve oral health and reduce inequities unless accompanied by upstream policies influencing macro‐level factors. Evidence of macro‐level or country‐level social determinants on oral health in paediatric populations is still scarce.^5^, ^6^, ^7^, ^8^ Understanding these determinants and their mechanisms of correlation might support the application of efficient and effective upstream oral health policies and prevention strategies.^9^ A better understanding of the socioeconomic context of oral health might allow to support population‐based preventive strategies and address unmet oral healthcare needs with adequately targeted interventions tailored according to the population needs and the characteristics of the local healthcare system.

Recent evidence reported the higher overall prevalence of oral disease in South European countries such as Croatia (60.6%) and Slovenia (58.6%), comparing with northern European countries (≈44%).^10^ Although caries prevalence trends declined in developed European countries, during the last couple of decades, stark inequalities are observed putting at‐risk population in less developed countries in southern and eastern parts of Europe, and also socially and economically deprived population groups in developed countries. Moreover, persons who are socially and economically disadvantaged have poor oral health accessibility and outcomes, since they tend to seeking urgent, not preventive dental treatment, which contributes to the already existed inequalities.

Previous categorization of healthcare systems according to models, such as Bismarck, Beveridge, Semashko, and Mediterranean, could not be applicable nowadays since each country's healthcare system and it is financing significantly vary according to local circumstances in order to apply a unique approach that ensures health services and financial protection in a local setting. Healthcare system categorization is very difficult, and there are still efforts to categorize, and assess similarities, differences, and performance assessment internationally. Moreover, this debate is still ongoing in scientific areas of health services, health economy, political sciences, epidemiology, and sociological realms. When analyzing dental care in European children, paediatric population usually has a broader coverage and more benefits than adults, but there is a variety regarding co‐payments, and the services covered.^11^ More research and understanding are needed in the area of international comparison of healthcare delivery, access, and coverage, in order to design targeted and efficient interventions to improve health and reduce unmet needs and oral health inequalities.

This study was planned to assess and evaluate the fluctuation of the caries prevalence in 12‐year‐olds in the European region and to assess the influence of socioeconomic contexts of each country, that is, gross national income (GNI), United Nations Statistical Division geographical categorization of European countries, unemployment rate, Human Development Index (HDI), and per capita expenditure on dental health care on the prevalence of caries in 12‐year‐olds.

## 2 MATERIALS AND METHODS

### 2.1 Study design and search strategy and data source

The present survey was designed as a systematic review. The search involved the PubMed, Scopus, Embase, and Google Scholar databases. The search was performed in June 2023 using keywords “dental,” “caries,” “permanent teeth,” “12‐years‐old,” and “Europe.” The reporting of this review followed the Preferred Reporting Items for Systematic Reviews and Meta‐Analyses (PRISMA) guideline (the PRISMA checklist is reported in the supplementary file, Table S1). The review protocol was registered (ID: 349408) in the International Prospective Register of Systematic Reviews (PROSPERO) system (https://eur03.safelinks.protection.outlook.com/?url=https%3A%2F%2Fwww.crd.york.ac.uk%2Fprospero%2Fdisplay_record.php%3FRecordID%3D349408&data=05%7C01%7Cguglielmo.campus%40unibe.ch%7Cde6f54c55ca44ce553f508dbdb6e24f7%7Cd400387a212f43eaac7f77aa12d7977e%7C1%7C0%7C638345038345429484%7CUnknown%7CTWFpbGZsb3d8eyJWIjoiMC4wLjAwMDAiLCJQIjoiV2luMzIiLCJBTiI6Ik1haWwiLCJXVCI6Mn0%3D%7C3000%7C%7C%7C&sdata=iroGwzmCU5VifWBD84uKY7ARrLbeSDnka%2F%2Bh5zNxIr8%3D&reserved=0).

Finally, data collected from scientific reports after searching the scientific databases and retrieving publications were from 36 countries of the European region (Table 1).

**TABLE 1:** Description of the different countries included, by socioeconomic indicators and healthcare systems. Countries are listed in alphabetical order.

Country	Income category^a^	United Nations Statistical Division geographical categorization of European countries (M49)	Unemployment^b^	Human Development Index^c^
Austria^13^	Very high	Western Europe	Medium	Very high
Albania^28^	Middle	Southern Europe	High	High
Belarus^29^	Middle	Eastern Europe	High	Very high
Belgium^13^	Very high	Western Europe	Medium	Very high
Bosnia and Herzegovina^30^	Middle	Southern Europe	High	High
Bulgaria^31^	Middle	Eastern Europe	Medium	Very high
Croatia^32^	Middle	Southern Europe	Medium	Very high
Cyprus^33^	Very high	Southern Europe	Medium	Very high
Denmark^13^	Very high	Northern Europe	Medium	Very high
Estonia^13^	High	Northern Europe	Medium	Very high
Finland^13^	Very high	Northern Europe	Medium	Very high
France^13^	Very high	Western Europe	High	Very high
Georgia^34^	Middle	Eastern Europe	High	Very high
Germany^35^, ^36^, ^37^	Very high	Western Europe	Low	Very high
Greece^38^, ^39^	High	Southern Europe	High	Very high
Greenland^40^	Very high	Northern Europe	Low	Very high
Hungary^41^	Middle	Eastern Europe	Low	High
Italy^42^	Very high	Southern Europe	Medium	Very high
Kosovo^43^	Middle	Southern Europe	High	High
Latvia^44^, ^45^	High	Northern Europe	Medium	Very high
Lithuania^46^	High	Northern Europe	Medium	Very high
Moldova^47^	Middle	Eastern Europe	Low	High
North Macedonia^48^, ^49^	Middle	Southern Europe	High	High
Norway^50^, ^51^	Very high	Northern Europe	Low	Very high
Poland^52^, ^53^, ^54^	High	Eastern Europe	Low	Very high
Portugal^55^	High	Southern Europe	Medium	Very high
Romania^23^, ^26^, ^27^	Middle	Eastern Europe	Medium	Very high
Russian Federeation^56^, ^57^, ^58^, ^59^	Middle	Eastern Europe	Low	Very high
Serbia^60^	Middle	Southern Europe	Low	Very high
Slovakia^61^, ^62^	High	Eastern Europe	Medium	Very high
Slovenia^63^	Very high	Southern Europe	Medium	Very high
Spain^64^, ^65^, ^66^	Very high	Southern Europe	High	Very high
Sweden^24^, ^25^	Very high	Northern Europe	Medium	Very high
Switzerland^21^, ^67^	Very high	Western Europe	Low	Very high
United Kingdom^68^, ^69^, ^70^, ^71^	Very high	Northern Europe	Low	Very high
Ukraine^72^	Middle	Eastern Europe	Medium	High

^a^ Low‐middle and low‐income countries with a GNI of $15 000 or less; the upper middle‐income countries with a GNI between $15 001 and $25 000; high‐income countries with a GNI of more than $25 000.

^b^ Low: countries with low unemployment rate of ≤5%; medium: countries with medium unemployment rate of more than 5% but ≤10%; high: countries with high unemployment rate of >10%.

^c^ High human development with HDI between 0.700 and 0.799; very high human development with a HDI of 0.800 or greater.

### 2.2 Eligibility criteria

**FIGURE 1:**
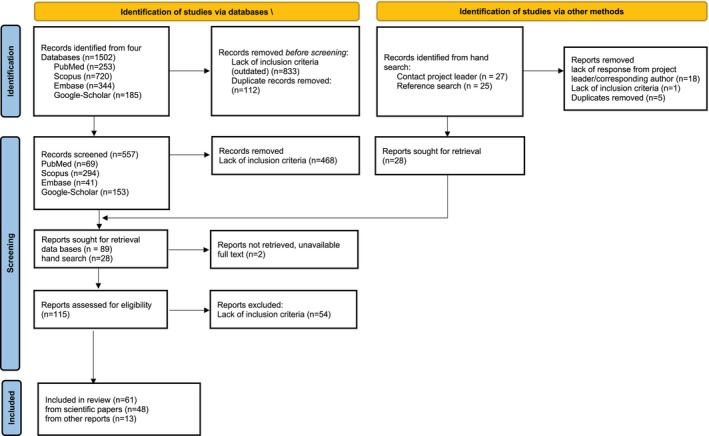
Preferred Reporting Items for Systematic Reviews and Meta‐Analyses (PRISMA) flowchart of the review process.

The inclusion criteria concerned observational epidemiological studies, cross‐sectional surveys, and dataset public health documents (national statistical oral health registers) dated within the period from 2011 to 2022 involving subjects aged 12 years old (Figure 1). Missing data were supplemented by contacting the corresponding authors. Moreover, the data were supplemented with the data obtained from master and/or doctoral theses and public health documents written in native national languages by contacting the corresponding authors. Publications from countries within the geographic region of Europe according to the United Nations Statistical Division were involved in this survey.^12^

The age of 12 represents an age group indicator in international epidemiological comparisons; as in most countries, these children are the oldest group attending primary schools having a full permanent dentition. Therefore, this age group is convenient for sampling and preventive public health intervention through school systems.

The exclusion criteria involved papers that were not possible to retrieve, surveys that involved sample subjects with special care needs, from regions outside of geographical region of Europe, before 2011, unfit age groups, and if results did not present caries experience as DMFT or when it was not possible to calculate D_3_MFT. Data below D_3_MFT scores were not used for the purposes of this survey.

### 2.3 Outcome Variables

All data of the participants were extracted by two authors (AV and KAS) from the literature search and, if needed, standardized for comparative purposes. The data sources used for obtaining information on oral health outcomes included scientific databases (PubMed, Scopus, Embase, and Google Scholar) and hand search using references and the Malmo University Database.^13^ The first database was designed in Excel Microsoft Office®. The DMFT was categorized as low experience with a DMFT ≤1, as medium‐high experience with a DMFT >1 but ≤2, as high experience with a DMFT >2 but ≤3, and as very high experience with a DMFT >3 by the authors, partially following the WHO handbook. After completion of data extraction, one author (KAS) randomly selected 10% of the papers and checked each data entry fields (especially data used for the metanalyses) to assess whether data extraction was carried out correctly.

### 2.4 Independent variables

The following socioeconomic indicators were added: GNI, United Nations Statistical Division geographical categorization of European countries (M49), unemployment rate, HDI, and per capita expenditure on dental health care.

The data on GNI were obtained via the World Bank Open Data sets, and there were no low‐income countries in the sample of this study (according to the World Bank, GNI is $1135 or less).^14^ Also, only three countries involved in the present survey could be categorized as lower middle income (according to the World Bank, GNI is between $1136 and $4465). More than half of all countries (*n* = 18) that were included in this survey could be categorized as high income (according to the World Bank, GNI is more than $13 846) ranging from Romania (GNI = $14 160) to Switzerland (GNI = $87 720). With this in mind and the distribution of GNI across European countries, the authors decided to modify World Bank categorization and apply it to the present survey using the following three income categories according to GNI: (1) middle income with a GNI of $15 000 or less; (2) high income with a GNI between $15 001 and $25 000; and (3) very high income with a GNI of more than $25 000 (Table 1). The mean number of decayed, missing, and filled teeth and the DMFT indices of the countries were then stratified according to the GNI category.

The countries were grouped according to the United Nations Statistical Division geographical categorization of European countries as follows: Eastern Europe, Northern Europe, Southern Europe and Western Europe (presented in Table 1).^12^

Data on the unemployment rate were obtained via World Bank Open Data and having in mind mean value and ranges of unemployment rate in the region of Europe the authors decided to categorize unemployment rate into following categories: countries with low unemployment rate of ≤5%; countries with medium unemployment rate of more than 5% but ≤10%; and countries with high unemployment rate of >10%.^15^

Although HDI involves four categories (low, medium, high, and very high), all subjects in this study comprised only two HDI categories: high human development with HDI between 0.700 and 0.799, and very high human development with a HDI of 0.800 or higher. These data were obtained via online available resources.^16^

Data on country per capita expenditure on dental health care (USD) were obtained through Organization for Economic Co‐operation and Development (OECD) e‐library.^17^

Quality assessment was performed using the customized quality assessment tool developed by The National Heart, Lung and Blood Institute for Observational Cohort and Cross‐sectional studies, case–control studies, and controlled intervention studies (https://www.nhlbi.nih.gov/health‐topics/study‐quality‐assessment‐tools). The quality of the papers was recorded according to the following scores: 11–14: good quality, 7–10: fair quality, and 0–6: poor quality.

### 2.5 Statistical methods

One‐way analysis of variance was run to evaluate whether the difference in caries experience stratified by geographical categorization, and socioeconomic indicators was statistically significant. Furthermore, the distribution of DMFT indices was compared with the previously described GNI classes. Fisher's exact test Yates Continuity Correction was performed to verify whether there is a statistically significant association between DMFT categories and the GNI.

Moreover, an ordinal logistic regression analysis of DMFT categories according to socioeconomic indicators and geographical country location was performed.

Lastly, a meta‐analysis was performed for countries that reported data on the sample size, mean, and standard deviation of the DMFT. If standard deviation was not reported within the publication, in order to be able to pool the effect in the meta‐analysis model it was either calculated from the available data or imputed, and additional sensitivity analysis was performed in that case.^18^ The standard deviation was converted to the standard error for the purpose of the meta‐analysis. If multiple reports were retrieved from a country, for the purposes of meta‐analysis the different reports were weighted, if the data were similar. If a single‐center/local/regional study and a nationwide study were retrieved from the same country, only the nationwide study was included. If there were only single‐center/local/regional studies available, the recent one was included in the analysis. The meta‐analysis of the mean DMFT was stratified by GNI and geographical location of the country, using a random‐effects model with 95% confidence interval. Heterogeneity between studies was reported using the *I* ^2^ statistic and a 95% prediction interval. To investigate heterogeneity, meta‐regression was performed for year of publication. Sensitivity analysis was performed to test the robustness of the results by running the meta‐analysis only with data obtained from national surveys and data in which the standard deviation was reported in the included studies. The meta‐analyses were performed using *meta* command in StataSE18®.

## 3 RESULTS

Total data included 493 360 children from samples of 61 scientific and public health reports in the 36 countries of the European region, ranging from 22 subjects in Belgium to 89 442 subjects in a study sample in United Kingdom.

Of 1502 records identified via four database search, 27 authors were contacted, and finally, 61 reports were analyzed (Figure 1).

The majority of the studies (*n* = 32, 56.15%) were classified as being of fair quality. Seventeen studies (33.33%) and two studies were ranked as being of poor and good quality, respectively (Appendix S1). Lack of sample size justification or power analysis (Question 5 in the quality assessment methods) affected the quality outcome of the papers. The data extraction agreement among examiners was high (93.5%). The quality assessments table is reported in Appendix (Table S2).

The DMFT index showed a statistically significant association (*p* < .01) with the countries' GNI (Table 2). The results revealed a highly statistically significant lower experience of decayed, missing, and/or filled teeth in higher‐income countries. A strong statistically significant association between income category and DMFT categories was noted: the lower the income, the higher the caries experience is (Table 2).

**TABLE 2:** DMFT index across European countries stratified by gross national income (GNI) categories^a^.

(A) Mean, standard deviation, and range				
Income category (GNI)	Decayed teeth, mean ± SD (range)	Missing teeth, mean ± SD (range)	Filled teeth, mean ± SD (range)	DMFT, mean ± SD (range)
Middle income	1.67 ± 0.55 (0.56–2.59)	—	1.00 (1.00–1.00)	3.17 ± 1.05 (1.82–6.88)
High income	1.21 ± 0.84 (0.33–2.10)	0.05 ± 0.04 (0.00–0.09)	1.09 ± 0.80 (0.52–2.00)	2.49 ± 0.99 (1.18–4.45)
Very high income	0.42 ± 0.73 (0.00–3.18)	0.13 ± 0.03 (0.00–0.05)	0.43 ± 0.23 (0.20–0.70)	0.85 ± 0.36 (0.30–2.03)
One‐way ANOVA	*F* = 10.84 *p* < .01	*F* = 1.29 *p* = .35	*F* = 2.23 *p* = .18	*F* = 53.23 *p* < **.01**

(B) DMFT categorization (low experience with a DMFT ≤1, as medium‐high experience with a DMFT >1 but ≤2, as high experience with a DMFT >2 but ≤3, and as very high experience with a DMFT >3)				
Income category (GNI)	Low experience, *n* (%)	Medium‐high experience, *n* (%)	High experience, *n* (%)	Very high experience, *n* (%)
Middle income	—	1 (5.00)	8 (40.00)	11 (55.00)
High income	—	5 (45.50)	3 (27.30)	3 (27.30)
Very high income	22 (81.50)	3 (11.10)	1 (3.70)	1 (3.70)

*Note*: Fisher's exact test with Yates continuity correction, *p* < .01. Number of countries and percentage. Statistically significant differences in bold.

^a^ Middle‐income countries: USD ≤15 000; high‐income countries: USD 15001/$25 000; very high‐income countries: USD >25 000.

Statistically significant differences in DT (*p* < .01) were observed according to the geographical location of the country (Table 3). Overall, the countries of West Europe presented the lowest experience of caries, followed by the countries in North Europe (*p* < .01). The East and South European countries clearly showed the highest experience of caries as shown in Table 3. The same features are observed when comparing the unemployment rate and DMFT (*p* < .05), as well as HDI with DMFT (*p* < .01; Table 3).

**TABLE 3:** DMFT index across European countries stratified by healthcare systems (A), unemployment rate (B) and Human Development Index (HDI) (C).

	Decayed teeth, mean ± SD (range)	Missing teeth, mean ± SD (range)	Filled teeth, mean ± SD (range)	DMFT, mean ± SD (range)
(a) M49 country categorization				
East Europe	1.86 ± 0.41 (1.54–2.59)	0.09 ± — (0.09–0.09)	0.52 ± — (0.52–0.52)	2.87 ± 0.53 (1.77–3.53)
South Europe	0.92 ± 0.66 (0.10–2.16)	0.03 ± 0.04 (0.00–0.06)	0.87 ± 0.18 (0.74–1.00)	2.31 ± 1.64 (0.65–6.88)
North Europe	0.99 ± 1.32 (0.00–3.18)	0.00 ± 0.00 (0.00–0.00)	0.80 ± 1.04 (0.20–2.00)	1.30 ± 1.14 (0.30–4.45)
West Europe	0.21 ± 0.16 (0.10–0.60)	0.02 ± 0.03 (0.00–0.05)	0.54 ± 0.18 (0.30–0.70)	0.80 ± 0.27 (0.44–1.36)
One‐way ANOVA	*F* = 5.62 ***p* < .01**	*F* = 2.69 *p* = .18	*F* = 0.19 *p* = .90	*F* = 9.62 ***p* < .01**
(b) Unemployment rate				
Low unemployment rate	0.83 ± 1.11 (0.00–3.18)	0.05 ± 0.04 (0.00–0.09)	0.42 ± 0.21 (0.20–0.67)	1.23 ± 0.89 (0.40–3.20)
Medium unemployment rate	0.87 ± 0.95 (0.00–2.59)	0.01 ± 0.03 (0.00–0.06)	0.83 ± 0.69 (0.20–2.00)	2.08 ± 1.23 (0.30–4.45)
High unemployment rate	1.07 ± 0.68 (0.10–2.16)	—	1.00 ± — (1.00–1.00)	2.38 ± 1.60 (0.65–6.88)
One‐way ANOVA	*F* = 0.24 *p* = .79	*F* = 0.89 *p* = .46	*F* = 0.82 *p* = .48	*F* = 3.30 ***p* < .05**
(c) Human Development Index				
High	1.62 ± 0.59 (0.56–2.16)	—	1.00 ± — (1.00–1.00)	3.41 ± −1.61 (1.82–6.88)
Very high	0.78 ± 0.88 (0.10–3.18)	0.03 ± 0.04 (0.00–0.09)	0.65 ± 0.55 (0.2–2.00)	1.73 ± 1.13 (0.30–4.45)
One‐way anova	*F* = 4.98 ***p* < .05**	*F* = 0.51 *p* = .50	*F* = 0.37 *p* = .56	*F* = 13.51 ***p* < .01**

*Note*: Statistically significant differences in bold.

A further analysis, using the ordinal logistic regression analysis, assessed the level of impact of socioeconomic determinants on caries experience in different countries. The results confirmed statistically significant differences between caries experience in countries categorized according to GNI of the country, with children living in higher‐income countries having 90% lower odds of poor oral health than children living in middle‐income countries (*p* < .01; Table 4). Although the strongest association was observed between countries' income category and caries experience, multinomial ordinal logistic regression analysis revealed that children living in Western European countries have 95% lower odds of oral disease than children living in the East or South of Europe (Table S3).

**TABLE 4:** Ordinal logistic regression analysis of the DMFT categorization by socioeconomic indicators and geographic location.

Variable	Odds ratio (SE)	*p*‐Value	95% confidence interval
DMFT categorization			
GNI	0.10 (0.07)	**.00**	0.03–0.37
Unemployment rate	0.99 (0.41)	.98	0.44–2.23
Human Development Index	1.82 (1.71)	.50	0.32–10.47
Geographical location	0.57 (0.25)	.20	0.24–1.35

*Note*: Number of observations = 58; LR *χ* ^2^_(4)_ = 53.99; log likelihood = −50.29; *p* < .01. Statistically significant associations in bold.

**FIGURE 2:**
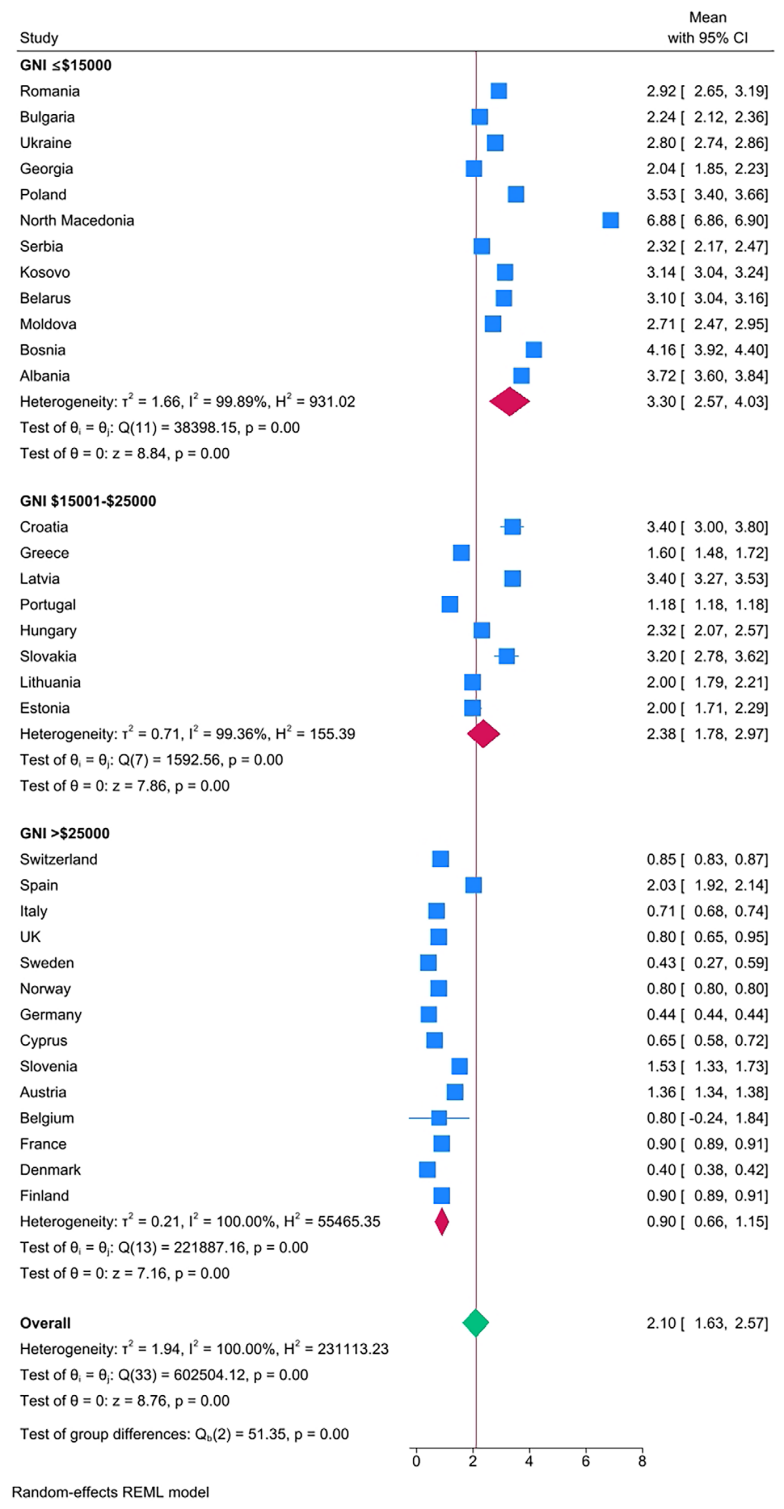
Forest plot of the pooled caries experience (DMFT) by gross national income (GNI) stratified by country and ordered by prevalence. Box size represents the sample size.

**FIGURE 3:**
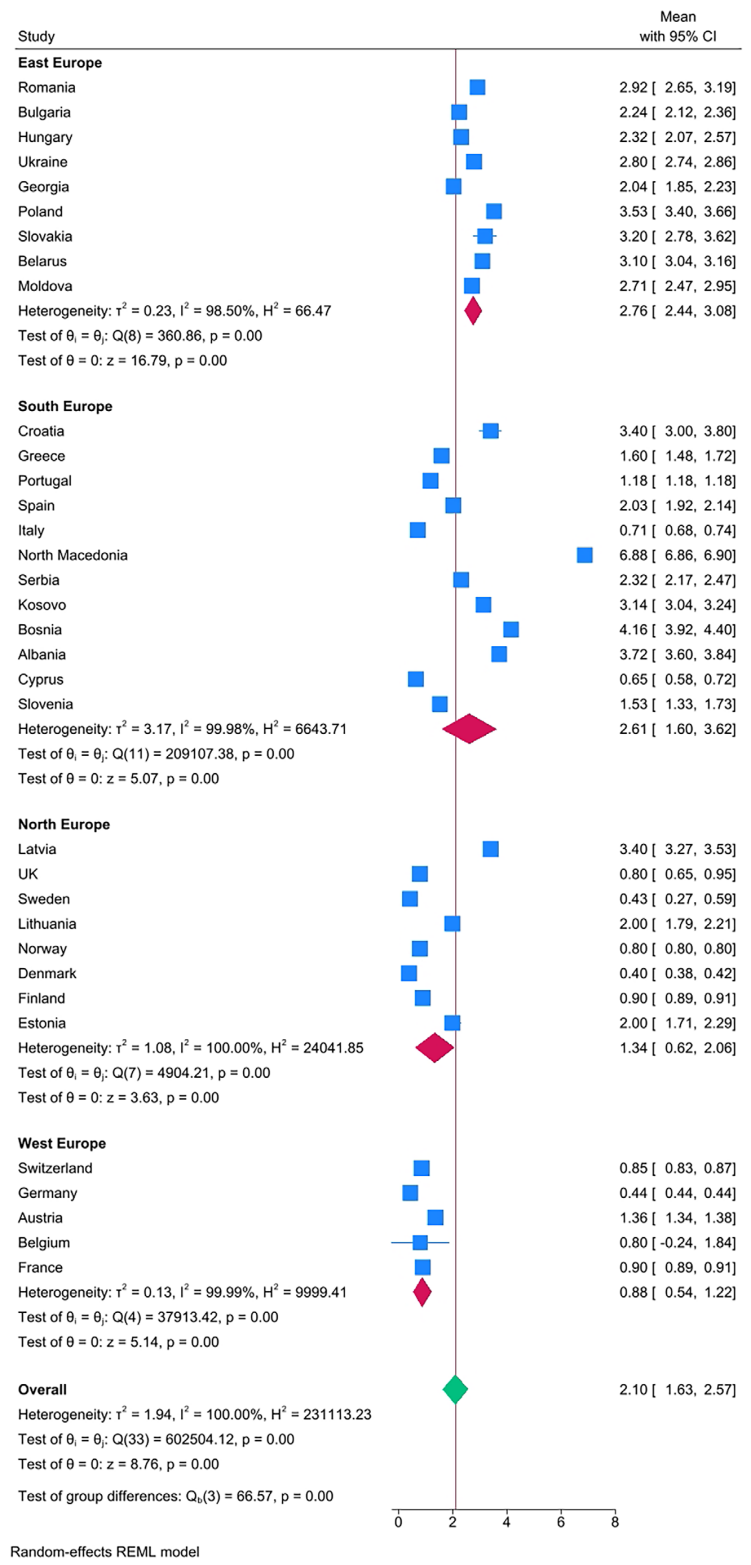
Forest plot of the pooled caries experience (DMFT) by geographical location stratified by country and ordered by prevalence. Box size represents the sample size.

**FIGURE 4:**
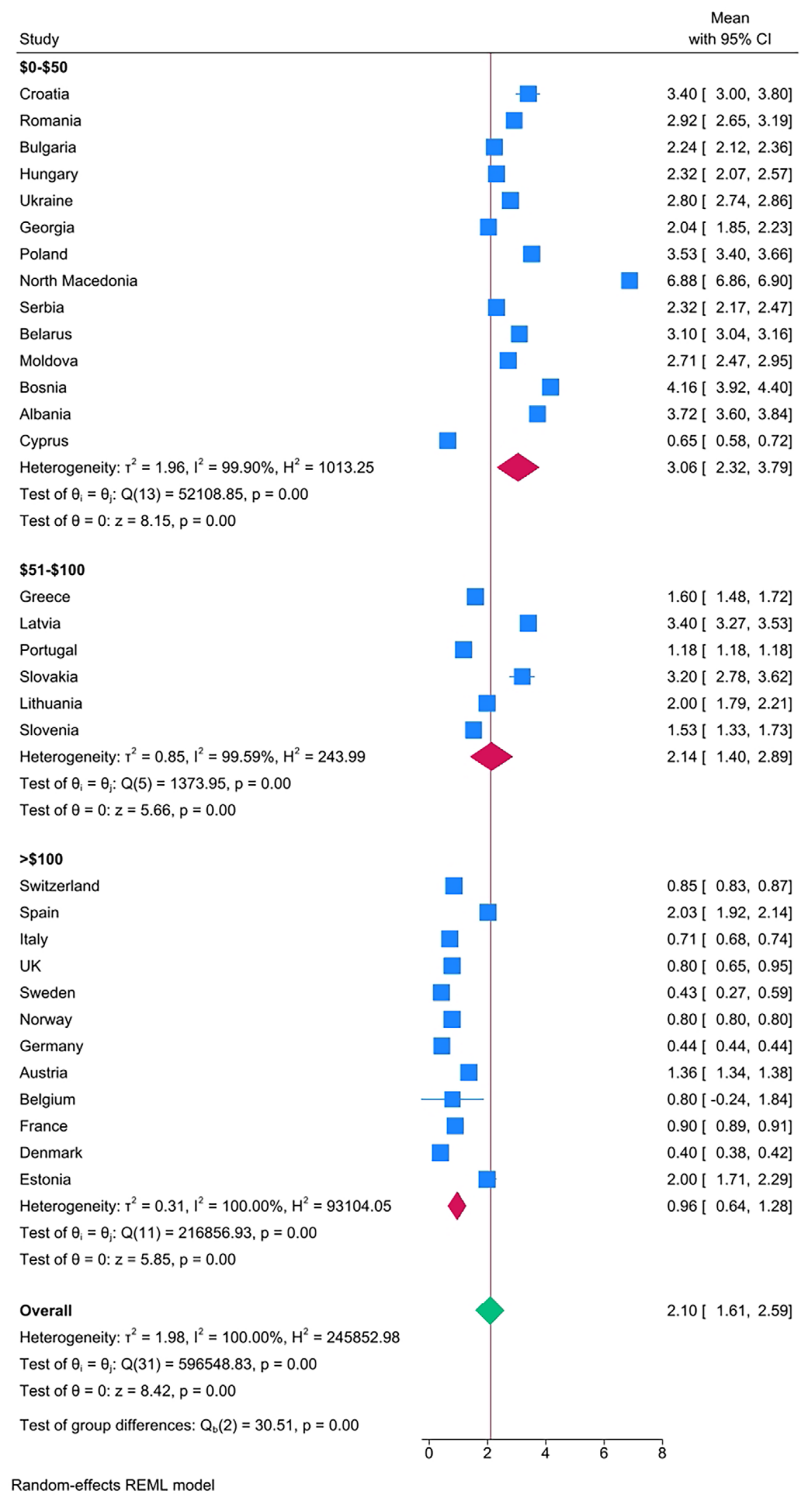
Forest plot of the pooled caries experience (DMFT) by per capita expenditure on dental health care stratified by country and ordered by prevalence. Box size represents the sample size.

The results confirmed clear differences in caries experience in Eastern European countries compared with Western European countries, when categorized according to both GNI (Figure 2) and geographical location (Figure 3). The overall mean DMFT by GNI category was 0.85 (GNI >25 000), 2.49 (GNI 15001–25 000), and 3.17 (GNI ≤$15 000), respectively. The overall DMFT according to the country geographical location was 0.80, 1.30, 2.31, and 2.87 for West Europe, North Europe, South European, and East Europe, respectively. Only 17% (*n* = 3) of the East European countries had a mean DMFT that was lower than the overall pooled mean DMFT of 2.10, whereas all the countries in the West Europe had a mean DMFT lower than the overall pooled mean DMFT. The extracted data were used to generate Figure 4 with 95% confidence intervals and test of significance within the mean DMFT of the subgroups of countries that were categorized according to per capita expenditure on dental health care—all countries with per capita expenditure on dental health above 100USD had lower mean DMFT than the overall pooled mean DMFT = 2.10. Meta‐regression results show that the year of publication and geographical location of the country explain 32% of in‐between study heterogeneity. Heterogeneity was high, reported at 100% with the *I* ^2^ statistic and a 95% prediction interval of 0.29–4.49 (Table S4). Sensitivity analysis results show that the overall pooled DMFT mean remained unchanged.

## 4 DISCUSSION

The results of the present survey showed that 12‐year‐old children living in economically disadvantaged European countries, and in particular in Eastern European countries present worse oral health conditions than children living in high‐income countries in northern part of Europe.

Although all countries included in this review are part of the European region, the dental health care of paediatric population revealed differences, mainly related to access, coverage, and benefits. Despite being broader compared with adults, the analysis of dental health care in children in Europe showed a variety regarding co‐payments, and the services covered, that diverge between different European states.^11^ Current debate in the dental public health scientific literature highlights the importance of social determinants of health on children's oral health. The provision of treatment‐focused modern dental care showed poor outcomes in meeting the populational needs, bearing in mind that the global numbers of untreated oral disease escalated by 1 billion during the last three decades.^19^ As caries exceeds any other NCD in prevalence, there is a need for radical change in prevention strategies, moving toward upstream public health solutions and addressing the underlying risk factors shared with other NCDs.^20^ Therefore, a better understanding and comparison of the different oral healthcare systems could provide more and clear information on their efficiency in the respective countries as well as why and how different factors influence oral health. Recent data gathered by the European Observatory on Health Systems and Polices confirmed that in most European countries, dental health care for children is almost fully covered, and children are mostly protected from the co‐payments; there are, however, so many differences and similarities in terms of funding, treatment coverage, age group considered, that the comparison according to the type of healthcare system is hardly possible.^11^

DMFT at 12 years of age is recognized as the leading indicator of the oral health in children and adolescents. The results of the present study confirmed that DMFT of children aged 12 years is statistically significantly higher in low/medium income countries, in countries in East and South Europe, and in countries with higher unemployment rates, up to DMFT value of 6.88.

Bearing in mind that oral health is considered an integral part of overall health and an important element of quality of life and well‐being, inequalities in oral health cannot be disregarded. Differences between countries are recognized by researchers, health professionals, politicians, and even lay people. Oral health inequalities are, however, completely avoidable, considering that oral diseases are mostly preventable and can be reduced and stopped with carefully planned preventive, prophylactic, and minimally invasive interventions. The same goes for immigrants or underprivileged children living in low socioeconomic circumstances. A preventive program carried out from 1964 to 2009 succeeded in reducing caries experience by 83% in the canton of Zurich, Switzerland.^21^ But as far as EU Members States were concerned, DMFT decreased at least twofold, up to sevenfold, when data from the mid‐70s were compared with the latest DMFT available at the beginning of the 21st century.^22^ Children living in Eastern Europe showed higher DMFT than those living in Western Europe, when data were available, but data unavailability presents an issue. The significant regional differences observed within the European continent were also confirmed by the poor availability of DMFT data in 12‐year‐old children in developing countries, confirming the poor monitoring, evaluation, and performance of healthcare systems, especially in the Balkan region compared with high‐income EU countries.^22^

The *p*‐value approaching 1, as presented in the figures, also explains *I* ^2^ = 0, suggesting high heterogeneity among all the included studies with regard to survey design and methodology. Similar methods for describing the oral health status of samples from different countries allow for comparing data and correctly interpreting of the results.

Nonetheless, taking into account the limitations of the present survey, the results should be interpreted carefully. Firstly, the survey covered a 10‐year period in which several countries' data (e.g., England, Wales and Northern Ireland, Greece, and Switzerland) were collected in 2012 or 2013—new health policies and political decisions may have been introduced in these years that could have changed the epidemiological situation in the respective countries. In addition, the COVID‐19 pandemic in 2020 has limited access to oral health care and stopped population oral health preventive programs worldwide, leading to a deterioration of oral health, especially in vulnerable social groups. Secondly, although most national caries data available for Europe include DMFT of 12‐year‐olds, it is very hard to compare these results having in mind differences in sampling methods and sample sizes. Studies assessing the caries experience in Belgium (*n* = 22),^13^ Romania (*n* = 99)^23^ and Sweden (*n* = 87)^24^ had rather smaller sample sizes (less than 100 subjects). This analysis, however, involved other studies from these countries having larger sample size.^25^, ^26^, ^27^ Moreover, it should be stressed that the sample size must always be considered in relation to the population size, country regions/districts, and social strata involved. The experience of the caries disease described in the surveys involving regional or local sampling should be carefully interpreted on a national level. The sensitivity analysis performed between regional and national data gathered for the purposes of this survey showed that the overall pooled DMFT mean remained unchanged. Moreover, considering that all studies evaluated involved cross‐sectional surveys, it is not possible to obtain precise information on cause and effect. Also, the present study did not include data on sugar consumption and the dental workforce in countries, which might be considered a limitation. Data describing caries, however, experience in the canton of Zurich over a 45‐year period did not observe any changes in sugar consumption in Switzerland from 1950 to 2009 although DMFT at the age of 12 years was reduced by 83%, indicating the effectiveness of school‐supervised toothbrushing programs.^20^ Lastly, social indicators for England, Wales, and Northern Ireland (GNI, unemployment rate, HDI) were used for the whole United Kingdom, although Scotland was not included in the assessment of caries experience in the present survey due to a lack of data for the last 10 years.

Although the authors obtained data on per capita expenditure on dental health care,^17^ these could not fit well in the regression model due to multicollinearity that was higher than *r* = .85, therefore influencing *p*‐value and statistical significance. This could be explained by the influence of each country's economic determinants on these data, affecting dental labor costs, used materials, technologies, and their costs.^11^ Nonetheless, as shown in Figure 4, the results confirmed strong and clear differences in caries experience in countries with different per capita expenditures on dental health care confirming lower DMFT values than pooled mean DMFT = 2.10 in countries with expenditures higher than 100USD. Further efforts and research should be carried out to explore in detail the influence of these variables on oral healthcare provision and internationally compare the results.

The present findings suggest a strong influence of the GNI, geographical location, unemployment rate, and state welfare on oral health. The literature search and meta‐analysis performed in the present survey confirmed a strong connection between oral disease and socioeconomic and political contexts. A better understanding of the concept of caries as a chronic NCD allows for novel approaches and preventive strategies that may prove to be more effective and cost‐efficient. Further research should emphasize a large‐scale sample involving larger observation periods and countries on all continents. Information on the unavailability of data, indicating poor monitoring and evaluation of preventive strategies, when they exist, would also be relevant.

This survey highlights the need to strengthen preventive strategies in European countries in transition with difficulties in financing oral health systems. Bearing in mind the strong effect of macro‐level, socioeconomic, political contexts on children's oral health, these findings strongly suggested the use of upstream approaches in the creation of preventive strategies for oral health. Inequalities in oral health are unfair and completely avoidable with policies that allow the allocation of appropriate resources in European countries.

## AUTHOR CONTRIBUTIONS

GC, KAS, and TGW conceived the ideas; KAS, AV, FC, RSR, RJ, AM, JFCR, MM, CM, AA, and MGC collected the data; GC, PC, AA, and RBB analyzed the data; AV, GC, MD, and MEO led the writing; AV, GC, MGC, MD, and MEO revised the paper and gave final approval of the manuscript.

## FUNDING INFORMATION

This research received no specific grant from any funding agency in the public, commercial, or not‐for‐profit sectors.

## CONFLICT OF INTEREST STATEMENT

None.

## Supporting information


Appendix S1.

## Data Availability

The data that support the findings of this study are available from the corresponding author upon reasonable request.
