# Supplementary material for: Caries status in 12‐year‐old children, geographical location and socioeconomic conditions across European countries: A systematic review and meta‐analysis
Source: Int J Paediatr Dent. 2024 Jun 16;35(1):201–15. doi: 10.1111/ipd.13224 (PMC11626496; doi:10.1111/ipd.13224)
Supplement: Supplementary file 1 — Appendix S1. [file IPD-35-201-s001.docx]

**SUPPLEMENTARY MATERIAL**

**“Caries status in 12-year-old children, health care systems and socio-economic conditions across European Countries. A systematic review and meta-analysis.”**

**Content**

Appendix Table 1. Prisma check list **Page 2**

Appendix Table 2. Quality assessment of the included papers **Page 4**

Appendix Table 3 Multinomial ordinal logistic regression analysis of the DMFT

categorization by socio-economic indicators and geographic location **Page 6**

Appendix Table 4. Output of the meta-regression analysis **Page 7**

Appendix Table 1. Prisma check list

| **Section and Topic** | **Item #** | **Checklist item** | **Location where item is reported** |
| --- | --- | --- | --- |
| **TITLE** | | |  |
| Title | 1 | Identify the report as a systematic review. | 1 |
| **ABSTRACT** | | |  |
| Abstract | 2 | See the PRISMA 2020 for Abstracts checklist. | 2 |
| **INTRODUCTION** | | |  |
| Rationale | 3 | Describe the rationale for the review in the context of existing knowledge. | 3 |
| Objectives | 4 | Provide an explicit statement of the objective(s) or question(s) the review addresses. | 3 |
| **METHODS** | | |  |
| Eligibility criteria | 5 | Specify the inclusion and exclusion criteria for the review and how studies were grouped for the syntheses. | 4,5 |
| Information sources | 6 | Specify all databases, registers, websites, organisations, reference lists and other sources searched or consulted to identify studies. Specify the date when each source was last searched or consulted. | 4 |
| Search strategy | 7 | Present the full search strategies for all databases, registers and websites, including any filters and limits used. | 4 |
| Selection process | 8 | Specify the methods used to decide whether a study met the inclusion criteria of the review, including how many reviewers screened each record and each report retrieved, whether they worked independently, and if applicable, details of automation tools used in the process. | 4,5 |
| Data collection process | 9 | Specify the methods used to collect data from reports, including how many reviewers collected data from each report, whether they worked independently, any processes for obtaining or confirming data from study investigators, and if applicable, details of automation tools used in the process. | 5 |
| Data items | 10a | List and define all outcomes for which data were sought. Specify whether all results that were compatible with each outcome domain in each study were sought (e.g. for all measures, time points, analyses), and if not, the methods used to decide which results to collect. | 5 |
|  | 10b | List and define all other variables for which data were sought (e.g. participant and intervention characteristics, funding sources). Describe any assumptions made about any missing or unclear information. | 6 |
| Study risk of bias assessment | 11 | Specify the methods used to assess risk of bias in the included studies, including details of the tool(s) used, how many reviewers assessed each study and whether they worked independently, and if applicable, details of automation tools used in the process. | 5 |
| Effect measures | 12 | Specify for each outcome the effect measure(s) (e.g. risk ratio, mean difference) used in the synthesis or presentation of results. | 5,6 |
| Synthesis methods | 13a | Describe the processes used to decide which studies were eligible for each synthesis (e.g. tabulating the study intervention characteristics and comparing against the planned groups for each synthesis (item #5)). | 5 |
|  | 13b | Describe any methods required to prepare the data for presentation or synthesis, such as handling of missing summary statistics, or data conversions. | 5 |
|  | 13c | Describe any methods used to tabulate or visually display results of individual studies and syntheses. | 5 |
|  | 13d | Describe any methods used to synthesize results and provide a rationale for the choice(s). If meta-analysis was performed, describe the model(s), method(s) to identify the presence and extent of statistical heterogeneity, and software package(s) used. | 5 |
|  | 13e | Describe any methods used to explore possible causes of heterogeneity among study results (e.g. subgroup analysis, meta-regression). | 5 |
|  | 13f | Describe any sensitivity analyses conducted to assess robustness of the synthesized results. | 5,6 |
| Reporting bias assessment | 14 | Describe any methods used to assess risk of bias due to missing results in a synthesis (arising from reporting biases). | 5,6 |
| Certainty assessment | 15 | Describe any methods used to assess certainty (or confidence) in the body of evidence for an outcome. | 5,6 |
| **RESULTS** | | |  |
| Study selection | 16a | Describe the results of the search and selection process, from the number of records identified in the search to the number of studies included in the review, ideally using a flow diagram. | 7 |
|  | 16b | Cite studies that might appear to meet the inclusion criteria, but which were excluded, and explain why they were excluded. | 7 |
| Study characteristics | 17 | Cite each included study and present its characteristics. | 7 |
| Risk of bias in studies | 18 | Present assessments of risk of bias for each included study. | 8 |
| Results of individual studies | 19 | For all outcomes, present, for each study: (a) summary statistics for each group (where appropriate) and (b) an effect estimate and its precision (e.g. confidence/credible interval), ideally using structured tables or plots. | 8 |
| Results of syntheses | 20a | For each synthesis, briefly summarise the characteristics and risk of bias among contributing studies. | 8,9 |
|  | 20b | Present results of all statistical syntheses conducted. If meta-analysis was done, present for each the summary estimate and its precision (e.g. confidence/credible interval) and measures of statistical heterogeneity. If comparing groups, describe the direction of the effect. | 8,9 |
|  | 20c | Present results of all investigations of possible causes of heterogeneity among study results. | 8 |
|  | 20d | Present results of all sensitivity analyses conducted to assess the robustness of the synthesized results. | 8 |
| Reporting biases | 21 | Present assessments of risk of bias due to missing results (arising from reporting biases) for each synthesis assessed. | 8,9 |
| Certainty of evidence | 22 | Present assessments of certainty (or confidence) in the body of evidence for each outcome assessed. | 8,9 |
| **DISCUSSION** | | |  |
| Discussion | 23a | Provide a general interpretation of the results in the context of other evidence. | 10 |
|  | 23b | Discuss any limitations of the evidence included in the review. | 11 |
|  | 23c | Discuss any limitations of the review processes used. | 11 |
|  | 23d | Discuss implications of the results for practice, policy, and future research. | 11,12 |
| **OTHER INFORMATION** | | |  |
| Registration and protocol | 24a | Provide registration information for the review, including register name and registration number, or state that the review was not registered. | 3 |
|  | 24b | Indicate where the review protocol can be accessed, or state that a protocol was not prepared. | 3 |
|  | 24c | Describe and explain any amendments to information provided at registration or in the protocol. | 3 |
| Support | 25 | Describe sources of financial or non-financial support for the review, and the role of the funders or sponsors in the review. | 13 |
| Competing interests | 26 | Declare any competing interests of review authors. | 13 |
| Availability of data, code and other materials | 27 | Report which of the following are publicly available and where they can be found: template data collection forms; data extracted from included studies; data used for all analyses; analytic code; any other materials used in the review. |  |

Appendix Table 2. Quality assessment of the included papers except national surveys not published (tool developed by The National Heart, Lung and Blood Institute for Observational Cohort and Cross-sectional studies, Case-Control studies and Controlled-Intervention studies (<https://www.nhlbi.nih.gov/health-topics/study-quality-assessment-tools>).

| Study ID | Title | Source | Country | Quality Assessment |
| --- | --- | --- | --- | --- |
| Hysi et al., 2014 | Caries experience and treatment needs among Albanian 12-year-olds. | Community Dent Health. 2014;31(3):141-144. | Albania | Fair |
| Leous, 2016 | The feasibility of descriptive epidemiology in assessments of dental caries disease in children in Russia and Belarus. | Stomatologiya. 2016;95(4):21. | Belarus | Poor |
| Markovic et al., 2013 | Oral Health in Bosnia and Herzegovina Schoolchildren - Findings of First National Survey | Austin J Dent. 2014;1(2):1010 | Bosnia | Poor |
| Zukanović, 2013 | Caries risk assessment models in caries prediction. | Acta Med Acad. 2013;42(2):198-208. doi:10.5644/ama2006-124.87 | Bosnia | Poor |
| Onov & Beltcheva, 2020 | Caries Prevalence in 12-year-old Children from Plovdiv - a Multifactorial Regression Analysis. | Folia Med (Plovdiv). 2020;62(1):159-164. doi:10.3897/folmed.62.e47894 | Bulgaria | Poor |
| Lešić et al, 2019 | Caries prevalence among schoolchildren in urban and rural Croatia. | Cent Eur J Public Health. 2019;27(3):256-262. doi:10.21101/cejph.a5314 | Croatia | Poor |
| Panagidis & Schulte, 2012 | Caries prevalence in 12-year-old Cypriot children.  20. | Community Dent Health. 2012;29(4):297-301. | Cyprus | Fair |
| Sgan-Cohen et al., 2014 | Dental caries among children in Georgia by age, gender, residence location and ethnic group. | Community Dent Health. 2014;31(3):163-166. | Georgia | Fair |
| Pieper et al., 2013 | K, Lange J, Jablonski-Momeni A, Schulte AG. Caries prevalence in 12-year-old children from Germany: results of the 2009 national survey. | Community Dent Health. 2013;30(3):138-142. | Germany | Fair |
| Jordan et al., 2014 | The Fifth German Oral Health Study (Fünfte Deutsche Mundgesundheitsstudie, DMS V) - rationale, design, and methods. | BMC Oral Health. 2014;14:161. | Germany | Fair |
| Splieth et al., 2019 | 40-Year Longitudinal Caries Development in German Adolescents in the Light of New Caries Measures. | Caries Res. 2019;53(6):609-616. | Germany | Good |
| Oulis et al., 2012 | Caries prevalence of 5, 12 and 15-year-old Greek children: a national pathfinder survey. | Community Dent Health. 2012;29(1):29-32. | Greece | Fair |
| Diamanti et al., 2021 | Oral hygiene and periodontal condition of 12- and 15-year-old Greek adolescents. Socio-behavioural risk indicators, self- rated oral health and changes in 10 years. | Eur J Paediatr Dent. 2021;22(2):98-106. | Greece | Fair |
| Ekstrand et al., 2020 | The impact of a national caries strategy in Greenland 10 years after implementation. A failure or a success? | Int J Circumpolar Health. 2020;79(1):1804260. | Greenland | Fair |
| Szöke et al., 2008 | Changing Levels of Dental Caries over 30 Years among Children in a Country of Central and Eastern Europe - The Case of Hungary. | Oral Health Prev Dent. 18(1):177-183 | Hungary | Fair |
| Campus et al., 2020 | Caries severity and socioeconomic inequalities in a nationwide setting: data from the Italian National pathfinder in 12-years children. | Sci Rep. 2020;10(1):15622. | Italy | Fair |
| Ferizi et al., 2020 | Oral Health Status Among 12-Year-Old Schoolchildren in Kosovo. | Pesqui Bras Odontopediatria Clin Integr. 2020;20. | Kosovo | Fair |
| Maldupa et al, 2021 | Caries Prevalence and Severity for 12-Year-Old Children in Latvia. | Int Dent J. 2021;71(3):214-223. | Latvia | Fair |
| Gudkina et al., 2016 | Factors influencing the caries experience of 6 and 12 year old children in Riga, Latvia. | Stomatologija. 2016;18(1):14-20. | Latvia | Poor |
| Narbutaitė et al., 2016 | Variation in fluorosis and caries experience among Lithuanian 12 year olds exposed to more than 1 ppm F in tap water. | J Investig Clin Dent. 2016;7(2):187-192. | Lithuania | Fair |
| Bilder et al., 2018 | The pathfinder study among schoolchildren in the Republic of Moldova: dental caries experience. | Int Dent J. 2018;68(5):344-347. | Moldova | Fair |
| Nonkulovski et al., 2022 | Dental caries experience of 12 year old children from Resen municipality.  35. | Journal of Dental Problems and Solutions. Published online January 19, 2022:001-005. | North Macedonia | Poor |
| Sarakinova et al., 2013 | National strategy for prevention of oral diseases in children from 0 to 14 years old age in the Republic of Macedonia for the period 2008-2018. | Pril (Makedon Akad Nauk Umet Odd Med Nauki). 2013;34(2):129-134. | North Macedonia | Poor |
| Sulo et al, 2022 | Regional variations in caries experience, predictors, and follow-up among children and adolescents in Western Norway. | Acta Odontol Scand. 2022;80(4):289-294. | Norway | Fair |
| Statistics Norway, 2021. | Dental Health Care 2015-2021. Available from: | https://www.ssb.no/en/statbank/table/11985/. | Norway | Fair |
| Olczak-Kowalczyk, 2016 | Dental caries level and sugar consumption in 12-year-old children from Poland. Advances in Clinical and Experimental. | Medicine. 2016;25(3):545-550. | Poland | Poor |
| Gaszynska et al., 2014 | Thirty years of evolution of oral health behaviours and dental caries in urban and rural areas in Poland. Annals of Agricultural and Environmental. | Medicine. 2014;21(3):557-561. | Poland | Poor |
| Rodakowska etal., 2013 | Epidemiological analysis of dental caries in 12-year-old children residing in urban and rural settings in the Podlaskie region of north-eastern Poland. | Ann Agric Environ Med. 2013;20(2):325-328. | Poland | Poor |
| Calado et al., 2017 | Caries prevalence and treatment needs in young people in Portugal: the third national study. | Community Dent Health. 2017;34(2):107-111. | Portugal | Fair |
| Veiga et al.,2015 | Prevalence of Dental Caries and Fissure Sealants in a Portuguese Sample of Adolescents. | PLoS One. 2015;10(3):e0121299. | Portugal | Fair |
| Sava-Rosianu et al., 2021 | Caries Prevalence Associated with Oral Health-Related Behaviors among Romanian Schoolchildren. doi:10.3390/ijerph18126515 | Int J Environ Res Public Health. 2021;18(12):6515. | Romania | Fair |
| Jipa et al., 2012 | Oral health status of children aged 6-12 years from the Danube Delta Biosphere Reserve. | Oral Health Dent Manag. 2012;11(1):39-45. | Romania | Poor |
| Ondine Lucaciu et al., 2020 | WHO Pathfinder Survey of Dental Caries in 6 and 12-Year Old Transylvanian Children and the Possible Correlation with Their Family Background, Oral-Health Behavior, and the Intake of Sweets. | Int J Environ Res Public Health. 2020;17(11):4180. | Romania | Fair |
| Leous et al, 2020 | Longitudinal study of the primary prevention effect on dental caries. | Stomatologiia (Mosk). 2020;99(2):26-33. | Russia | Poor |
| Smolyar & Chuhray, 2015 | The study of caries incidence in children according to WHO Significant Index of Caries. | Stomatologiya. 2015;94(6):41. | Russia | Poor |
| Peric et al, 2022 | Oral Health in 12- and 15-Year-Old Children in Serbia: A National Pathfinder Study. | Int J Environ Res Public Health. 2022;19(19). | Serbia | Fair |
| Pilát et al., 2020 | Oral health status of 6- and 12-year-old children of Roma origin from Eastern Slovakia: a pilot study. doi:10.21101/cejph.a6225 | Cent Eur J Public Health. 2020;28(4):292-296. | Slovakia | Fair |
| Vrbič et al., | Epidemiology of Dental Caries and Disease Prevention Among 12-Year-Olds in Slovenia Over Thirty Years (1987-2017). | Oral Health Prev Dent. 18(1):185-196. | Slovenia | Fair |
| Montero et al., 2016 | Oral health-related quality of life in 6- to 12-year-old schoolchildren in Spain. | Int J Paediatr Dent. 2016;26(3):220-230. | Spain | Fair |
| Obregón-Rodríguez et al., 2019 | Prevalence and caries-related risk factors in schoolchildren of 12- and 15-year-old: a cross-sectional study. | BMC Oral Health. 2019;19(1):120. | Spain | Fair |
| Almerich-Silla et al., 2010 | Caries Prevalence in Children from Valencia (Spain) using ICDAS II criteria. | 2010. Med Oral Patol Oral Cir Bucal. Published online 2014:e574-e580 | Spain | Fair |
| Bravo et al., 2020 | Encuesta de salud oral en España 2020. | Revista Del Ilustre Consejo General De Colegios De Odontólogos Y Estomatólogos De España. 2020;25(4):1-35. | Spain | Poor |
| Mensah et al., 2021 | Swedish quality registry for caries and periodontal diseases (SKaPa): validation of data on dental caries in 6- and 12-year-old children. | BMC Oral Health. 2021;21(1):373. | Sweden | Poor |
| Kramer et al., 2016 | Demographic factors and dental health of Swedish children and adolescents. | Acta Odontol Scand. 2016;74(3):178-185. | Sweden | Fair |
| Steiner et al., 2010 | Changes in dental caries in Zurich school-children over a period of 45 years. | Schweiz Monatsschr Zahnmed. 2010;120(12):1084-1104. | Switzerland | Fair |
| Waltimo et al., 2015 | Caries experience in 7-, 12-, and 15-year-old schoolchildren in the canton of Basel-Landschaft, Switzerland, from 1992 to 2011. | Community Dent Oral Epidemiol. 2015: | UK | Fair |
| Wang et al., 2021 | Dental caries thresholds among adolescents in England, Wales, and Northern Ireland, 2013 at 12, and 15 years: implications for epidemiology and clinical care. | BMC Oral Health. 2021;21(1):137. | UK | Fair |
| Vernazza et al., 2016 | Caries experience, the caries burden and associated factors in children in England, Wales and Northern Ireland 2013. | Br Dent J. 2016;221(6):315-320. | UK | Fair |
| Baker et al., 2018 | Structural Determinants and Children’s Oral Health: A Cross-National Study. doi:10.1177/0022034518767401 | J Dent Res. 2018;97(10):1129-1136. | UK | Good |
| Davies et al., 1995 | The caries experience of 11 to 12 year-old children in Scotland and Wales and 12 year-olds in England in 2008-2009: reports of coordinated surveys using BASCD methodology. | Community Dent Health. 2012;29(1):8-13. | UK | Fair |
| Trufanova et al., 2018 | Characteristics of epidemiology of dental caries in children from regions with high and optimum fluorine content in drinking water. | Wiad Lek. 2018;71(2 pt 2):335-338. | Ukraine | Poor |

Appendix Table 3. Multinomial ordinal logistic regression analysis of the DMFT categorization by socio-economic indicators and geographic location

*Number of observations =58 LR χ^2^_(11)_ = 64.48 Log likelihood= -43.05 p<0.01*

| **Variable** | **Relative Risk Ratio (SE)** | **P-value** | **95% Confidence Interval** |
| --- | --- | --- | --- |
| *DMFT Categorization* | | | |
| 1 |  |  |  |
| Unemployment rate | 0.50 (0.44) | 0.43 | 0.09 -2.80 |
| Geographical location | 0.23 (0.24) | 0.16 | 0.29 – 1.84 |
| 2 |  |  |  |
| Unemployment rate | 0.37 (0.38) | 0.33 | 0.05 -2.82 |
| Geographical location | 0.05 (0.06) | **0.02** | 0.00 – 0.62 |
| 3 |  |  |  |
| Unemployment rate | 0.37 (0.40) | 0.35 | 0.05 – 2.99 |
| Geographical location | 0.09 (0.11) | **0.05** | 0.01 -1.05 |

Appendix Table 4. Meta-regression analysis of the different health systems an publication years of the papers included.
